# Anæsthetics

**Published:** 1863-11

**Authors:** J. D. White


THE
DENTAL REGISTER OF THE WEST

VOL. XVII.] NOVEMBER, 1863. [No. 11.

### Original Essays and Communications.

### ANÆSTHETICS.

BY J. D. WHITE, D. D. S.

Read before the Pittsburgh Dental Association.

GENTLEMEN AND FELLOW MEMBERS :—In compliance with an appointment made by the President of this Association, I present to your kind consideration the few ideas that I have been able to gather in connection with the present subject.

Since the first surgical operation was performed, the object of all practitioners has been to avoid pain; with this idea in view, many have been the agents suggested to meet this important end. The earliest means in use we find in the writings of Theodoric, about the latter part of the thirteenth century; these consisted in thoroughly impregnating a sponge with a strong aqueous extract of various anodyne articles such as opium, hemlock, hyoscyamus, lettuce and mandragora; this was held to the nose of the patient until sleep was produced, and when it was desired to arouse him, another sponge dipped in vinegar was applied as the former: if this expedient failed, the juice of fenugreek was freely injected into the nostrils. Near the close of the last century, strong hopes were entertained that a successful agent had finally been discovered, in the inhalation of nitrade oxide gas, either alone, or variously combined with other vapors; but after numerous experiments, in which Sir Humphrey Davy and other eminent philosophers took an active part, the idea was finally abandoned as chimerical. In 1819, Mr. James Nardrop of London, proposed to diminish the pain of surgical operations by means of copious venesections. In a paper published on this subject in the tenth volume of the Medico-Chirurgical Transactions, several cases are cited where its beneficial results had been successful; notwithstanding, it met with but little favor. Between this time and the year 1842, one hundred and one remedies were applied; but about this time, Dr. Horace Wells, a dentist of Hartford, Conn., wishing to have a tooth extracted, inhaled nitrous oxide gas, rendering himself completely insensible to pain; he afterwards administered the same agent to a number of his patients with success. Subsequently he repeated his experiments before the medical faculty and students at Hartford University, at Boston, but unfortunately for this gentleman, through some mismanagement he failed, and his reward was ridicule. In the month of October, 1846, two years subsequent to this, Dr. Morton, a dentist of Boston, and formerly a pupil of Dr. Wells, inhaled sulphuric ether, and afterwards administered it to a number of his patients with marked success. Feeling that it could be taken with impunity and with a perfect certainty of preventing pain, he applied to Dr. Warren of the Mass. General Hospital, for the privilege of experimenting upon a patient then in that Institution, who was about to undergo an operation for the removal of tumor of the neck by Dr. Warren, the result was a success. The news of this remedy spread rapidly, and it soon became general in the United States and in Europe. If possible, more attention was paid to the subject in the latter country than in this, and soon led to the discovery of chloroform by Dr. Simpson, of Edinburgh, in November of the year 1847. It was brought forward by this gentleman as a substitute for ether, he claiming for it great advantages over ether in the smallness of its doses, in its more prompt action, in its more agreeable effects, etc. To avoid making this article lengthy, I will pass from sulphuric ether to chloroform; as their administration and effects are much the same, I will treat them under one head. Chloroform is a compound of 3 equ. of chlorine and 1 of formyl, and is therefore the ter-chloride of formyl. Again, it might be said to consist of 2 atoms of carbon, 1 of hydrogen, and 3 of chlorine, it is a clear, colorless liquid, very volatile, of an agreeable aromatic odor, of a pungent saccharine taste, very dense, and of a specific gravity of 1·496, almost insoluble in water, non-inflamable, perfectly neutral, neither coloring nor bleaching litmus paper. It was first discovered by Mr. Samms Guthrie, of Sackett's Harbor, N.Y., as early as 1831; but its true composition was first accurately determined by Dumas in 1835, by whom it was called chloroform, from its relation to formic acids, being that article with its 3 equ. of oxygen, replaced by 3 of chlorine. Like ether, chloroform is subject to many impurities; but among all the impurities which it is liable to contain, the chlorinated Pyrogenous oils are the most injurious; these are detected and removed by pure and strong sulphuric acid; they are easily set on fire, and burn with a smoky flame, chlorine being among the products of their combustion. When the vapor of these oils are inhaled, according to Dr. Gregory, it causes distressing sickness and headache. There are many tests by which they may be discovered; a very delicate one is, to pour some of the chloroform upon the hand, when it quickly evaporates, leaving the oily impurities recognizable, now that they are no longer disguised by the chloroform; if pure, it will leave little or no smell. If pure chloroform be dropped upon white paper, it will evaporate leaving no trace; it is a powerful antisceptic; when taken internally it acts as a sedative narcotic, acting probably through the nervous system, and through the medium of the blood. It has been detected by Ragsby in the blood, and by Dr. Snow, of London, in different parts of the body after death. It may be administered in three different ways: 1st, internally; 2d, externally; and 3d, by inhalation. The first case in which it was thus em ployed is related by Prof. Ives, of New Haven, as early as January 2d, 18[illegible]2; the case was one of pulmonic disease, and was effectually relieved. In 1847, Dr. Simpson brought it forward as a new remedy for the relief of pain by inhalation in surgical operations.

Practically it is of little consequence to know who was the discoverer of anæsthetics; though if we make a careful inquiry into the matter, we can not fail to award to Dr. Wells the credit of having first applied it successfully in the prevention of pain during surgical operations: and had his experiments not resulted in disappointment, there is no doubt he would have pushed his researches further and perhaps have discovered the very means which Drs. Morton and Simpson afterwards found to possess such valuable properties—to both these latter gentlemen, however, the world owes a debt of gratitude. Chloroform as an anæsthetic agent possesses superior advantages over ether; among the most important are—first, the rapid manifestation of the anæsthetic action of the remedy; second, the less liability to cause vomiting and other unpleasant effects; third, the smaller amount of laryngial and bronchial irritation; and lastly, the more easy maintenance of the influence after the system has once been properly affected. It certainly requires more caution in its administration than ether, from the fact that the rate of mortality from its use has already reached about one hundred, whereas the deaths from ether have been comparatively speaking but few. The Mass. General Hospital has prohibited the use of any other agent than ether in surgical operations in that institution. This may be accounted for, I think, from the fact that ether was first applied in surgical operations in that Hospital; but where this institution has condemned its use, we find others advocating it as superior to all other remedies in the relief of pain. In St. Bartholomew's Hospital, according to Mr. Skey, chloroform had been administered up to 1854, in 9,000 cases without a single accident; a fact which will go far to prove the contrary theory to the Mass. Hospital. In Guy's Hospital, in London, it was administered upwards of 12,000 times, before any accident occurred. In the Crimean war, according to M. Flourens, it was administered more than 25,000 times without a single death. It has been asserted by the opponents of chloroform, that the rate of mortality has been increased in the larger surgical operations since its introduction. Mr. Arnott, of London, (by the way the great advocate of local anæsthesia,) claims to have established this fact upon an irrefragible basis: on the other hand, Dr. Simpson, the discoverer of its anæsthetic properties, from elaborate statistics which he has collected, shows that the deaths are not increased, but absolutely diminished. Dr. M'Lloyd, in his note on the surgery of the war in the Crimea, declares it as his conscientious belief that many lives were saved, and many operations performed, that would not have been attempted had it not been for its assistance.

In its administration there are five circumstancs to which particular attention should be paid, they are, recumbency; an empty stomach; a free play of the diaphragm; an abundance of atmospheric air, and a gradual administration. 1st, the position of the patient should be recumbent, care should be taken to depress the head and shoulders sufficiently; during etherization this is not necessary, the patient may sit up with safety, owing perhaps to the less relaxation of the muscles, and consequently of the less difficulty in maintaining the circulation of the brain, through the heart's action. 2d, For two reasons we should all desire an empty state of the stomach,—1st, if chloroform be given upon a full stomach, vomiting will be very likely to occur, and secondly, a crowded state of the stomach may interfere materially with the action of the diaphragm, therefore it would be better if food had not been taken for at least four hours previous to its administration; the interval, however, should not be too long, for fear that nervous exhaustion result from a want of stimulation. 3d, Care should also be taken that the patient's clothing be sufficiently loose to prevent any interference of the chest or stomach ; should these parts become obstructed in their action, much trouble may occur. 4th, Get all the atmospheric air which you can possibly attain ; this is important, as it is absolutely necessary to the safety of your patient—in etherization this is of comparatively little consequence. 5th, Inhalation should not be conducted in a hurried manner, time should be given for the system to accommodate itself to the influence of the remedy, thus relieving it of the shock it might otherwise sustain : in case the patient should be feeble or timid, from a half ounce to an ounce of brandy might be given previous to inhalation. The most convenient inhaler is a handkerchief folded in the form of a bird's nest, or funnel shaped. Innumerable inhalers have been suggested, but all have been objectionable on account of the difficulty of obtaining a sufficiency of atmospheric air. The patient after having taken his seat in the chair, empties his lungs with a deep expiration ; the napkin impregnated with the anæsthetic is held over the mouth and nose, distant about two inches ; bring it gradually nearer until the distance is reduced to about half an inch, beyond this it should not go : this should be repeated until the patient is entirely insensible to surrounding objects. This condition is generally produced in from one to two minutes, and generally lasts from five to ten ; in this condition the muscles are relaxed, and are no longer under the control of the will, the limbs retaining any position in which they may be placed ; the eyes are closed, the balls upturned, the pupils contracted and insensible to light, respiration is calm and easy, pulse soft and undisturbed, every thing betokening the patient in a quiet sleep, unconscious of surrounding objects, and therefore free from pain. If this should be carried a little further, coma will arise ; the patient will commence to snore, the force and frequency of the pulse and respiration will diminish, the pupil will become dilated ; but as yet all is well—a few more whiffs and the patient is in eternity. Hence the necessity of avoiding this degree of anæsthesia ; all prudent operators should be content with an impression short of the total abolition of consciousness,—inhaled to an excess, it sometimes produces results from which the patient does not recover for days ; such as the deprivation of smell, the perversion of taste, and loss of power in the bladder and rectum.

Dr. Hoppoldt, of South Carolina, reports a case where these effects did not wear off for two months. What constitutes a dose of chloroform has been variously estimated; from half an ounce to an ounce has been generally regarded as a dose, but sometimes from two to five times this amount is required, according to the susceptibility of the patient and the duration of the operation. Age is no barrier to the use of anæsthetics, they have been given to very old persons and to children ; one case is on record, where Dr. Gross, Professor of Surgery in Jefferson College, Philadelphia, administered it to an infant under two months old, with happy results. In cases of children a very small quantity only is required, and that held a considerable distance from the nose. It has usually been considered unsafe to administer anæsthetics to persons laboring under certain diseases, particularly organic lesions of the heart and brain ; but this same gentleman (Dr. Gross), says he has never allowed any affection whatever to stand in his way, nor can he see upon any physiological principle why they should ; since by tranquilizing the system, they effectually prevent the mental and bodily perturbation, which is so apt to attend operations performed without the aid of these agents. An overdose may destroy life instantaneously, or death may occur even after the reaction has partially taken place. As to the manner in which death is produced by anæsthetization, it has never, I believe, been positively settled, though it is believed that the carbonic acid gas contained in the chloroform, acting primarily upon the blood and nervous centres, and secondly upon the respiratory organs, prevents the proper performance of their functional duties, producing death by asphyxia. The treatment for the relief of an overdose of chloroform must be very prompt; the first thing is, desist from further administration; second, draw the tongue out of the mouth so as to lift it off the glottis; third, cause full access of air by throwing open the doors and windows, and by a free use of the fan; fourth, dash freely upon the face, cold water; fifth, introduce artificial respiration by introducing a tube into the windpipe; sixth, stimulate the surface of the body with hot mustard, or diluted spirits of ammonia; and lastly, if necessary, apply the galvanic electricity. When the patient is able to swallow, strong coffee may be given with advantage, or a free use of brandy and ammonia. For the minor effects of chloroform, a discontinuance of the inhalation, a little water sprinkled upon the face, and a free access of air, is all that is generally necessary. If vomiting should occur, the patient should be turned upon their side, with the head inclined downward, not upon the stomach, lest the free action of the diaphragm be impeded—for etherization the same general rules may be resorted to.

Amalyn as an anæsthetic I know but little of, it was introduced first by Dr. Snow of London, in 1857; but owing to the great danger attending its use it has met with but little favor: two cases are on record where it proved fatal in the hands of Dr. Snow, after he had administered it successfully in 143 cases. The Academy of Medicine, of Paris, have condemned it, under the belief that it induces death by paralysis of the heart. It has its advantages over chloroform, in that it produces anæsthesia more rapidly and in smaller quantities; that it is less liable to cause vomiting, struggling, muscular rigidity, sickening of the stomach, or profound coma; the patient recovers from the effects in a few moments after its discontinuance; its alleged advantages, however, have not been found to counterbalance the danger of its use. It is prepared by distilling amalyn alcohol with a concentrated solution of chloride of zinc. With local anæsthesia I have had no experience, but so far as my researches have gone, I am satisfied its advantages have been greatly overrated: besides, the application is not without risk. With these remarks, gentlemen, I will close; and if I have benefitted any member present with even one idea, I am repaid.

